# Small-Conductance Ca^2+^-Activated K^+^ Channel 2 in the Dorsal Horn of Spinal Cord Participates in Visceral Hypersensitivity in Rats

**DOI:** 10.3389/fphar.2018.00840

**Published:** 2018-08-03

**Authors:** Yu Song, Jun-Sheng Zhu, Rong Hua, Lei Du, Si-Ting Huang, Robert W. Stackman, Gongliang Zhang, Yong-Mei Zhang

**Affiliations:** ^1^Jiangsu Province Key Laboratory of Anesthesiology, Xuzhou Medical University, Xuzhou, China; ^2^Emergency Department, The Affiliated Hospital of Xuzhou Medical University, Xuzhou, China; ^3^Department of Psychology, Florida Atlantic University, Boca Raton, FL, United States; ^4^College of Basic Medical Sciences, Anhui Medical University, Hefei, China

**Keywords:** small-conductance Ca^2+^-activated K^+^ channel 2, visceral hypersensitivity, colorectal distension, spinal dorsal horn, rats

## Abstract

Visceral hypersensitivity is a highly complex and subjective phenomenon associated with multiple levels of the nervous system and a wide range of neurotransmission. The dorsal horn (DH) in spinal cord relays the peripheral sensory information into the brain. Small conductance Ca^2+^-activated K^+^ (SK) channels regulate neuronal excitability and firing by allowing K^+^ to efflux in response to increase in the intracellular Ca^2+^ level. In this study, we examined the influence of SK2 channels in the spinal DH on the pathogenesis of visceral hypersensitivity induced by colorectal distension (CRD) in rats. Electrophysiological results showed that rats with visceral hypersensitivity presented a decrease in the SK channel-mediated afterhyperpolarization current (*I*_AHP_), and an increase in neuronal firing rates and *c*-Fos positive staining in the spinal DH. Western blot data revealed a decrease in the SK2 channel protein in the membrane fraction. Moreover, intrathecal administration of the SK2 channel activator 1-EBIO or CyPPA alleviated visceral hypersensitivity, reversed the decrease in *I*_AHP_ and the increase in neuronal firing rates in spinal DH in rats that experienced CRD. 1-EBIO or CyPPA effect could be prevented by SK2 channel blocker apamin. CRD induced an increase in *c*-Fos protein expression in the spinal DH, which was prevented by 1-EBIO. Together, these data suggest that visceral hypersensitivity and pain is associated with a decrease in the number and function of membrane SK2 channels in the spinal DH. Pharmacological manipulation of SK2 channels may open a new avenue for the treatment of visceral hypersensitivity and pain.

**Highlights:**

- Neonatal colorectal distension induced visceral hypersensitivity in rats.
- Visceral hypersensitivity rats presented a decrease in afterhyperpolarization current (*I*_AHP_) and membrane SK2 channel protein in the spinal dorsal horn.
- Visceral hypersensitivity rats presented an increase in neuronal firing rate in the spinal dorsal horn.
- Intrathecal administration of SK2 channel activator 1-EBIO or CyPPA prevented visceral hypersensitivity and decrease in *I*_AHP_.

## Introduction

Visceral hypersensitivity is a hallmark of irritable bowel syndrome and other gastrointestinal disorders that cause pain. The mechanism of visceral hypersensitivity and pain is still poorly understood due to the diverse nature of visceral hypersensitivity compounded by multiple factors such as sexual dimorphism, psychological and physical stresses, genetic and epigenetic traits, and the nature of predisposed disease (Chen et al., 2015; Elsenbruch and Enck, 2016). We recently demonstrated that early life stresses, for example, neonatal colorectal distension (CRD), maternal separation, could induce visceral hypersensitivity in rats (Wang et al., 2016; Zhang et al., 2016a,b). Visceral information is collected by thoracolumbar and sacral spinal visceral afferents, and coded by viscerosomatic neurons in spinal cord, thalamus, limbic system, and cortex (Larauche et al., 2012; Moloney et al., 2015). The dorsal horn (DH) in spinal cord is a major component of the nociceptive system, and the alteration in spinal neuronal activity may modulate visceral hypersensitivity and pain (Kawamata and Omote, 1996; Wang et al., 2014; Fu et al., 2016; Medrano et al., 2016).

Peripheral visceral afferents project into the spinal lamina I, II (outer layer), V, and X before reaching the intermediolateral nucleus where the preganglionic neurons are localized (Sugiura et al., 1989). Visceral hypersensitivity can be induced by the sensitization of primary sensory neurons innervating the visceral organs, hyperexcitability of spinal ascending neurons receiving visceral afferents, and/or dysregulation of descending pathways that modulate spinal nociceptive transmission (Sengupta, 2009). Previous studies revealed that nociceptive neurons in the spinal DH presented higher excitability and exaggerated sensory reflex to the visceral stimuli in subjects with visceral hypersensitivity (Lu et al., 2007; Hipolito et al., 2015; Wu et al., 2016). Neuronal excitability is modulated by membrane ion channels, for example, small conductance Ca^2+^-activated K^+^ (SK) channels (Adelman et al., 2012).

The SK channels (SK1, SK2, and SK3 channels) distribute extensively in the brain (Kohler et al., 1996; Deignan et al., 2012), spinal dorsal root ganglia (DRG) and DH neurons (Mongan et al., 2005). SK2 channels were localized almost exclusively in the superficial laminae of the spinal DH, a region in which many sensory afferents terminate (Sailer et al., 2004; Mongan et al., 2005). SK2 channels regulate neuronal excitability and firing by allowing K^+^ to efflux in response to increases in the intracellular Ca^2+^ level (Adelman et al., 2012). SK channels in the spinal cord are involved in nociception. For example, 1-EBIO, a SK channel opener, inhibits neuronal activity in the dorsal root (Bahia et al., 2005). The SK2/SK3 channel blocker UCL 1,848 increases DH neuronal responses to naturally evoked nociceptive stimuli, and intraplantar injection of the selective SK channel blocker, apamin, induces mechanical allodynia and heat hyperalgesia in naive rats (Pagadala et al., 2013). SK channels in the brain modulate pathologic pain (Thompson et al., 2015), however, the role of spinal SK2 channels on the etiology of visceral hypersensitivity and pain remains elusive. In this study, we adopted CRD to develop visceral hypersensitivity and examined the mechanism of SK2 channels on visceral hypersensitivity and pain by examining SK2 channel-mediated current change and SK2 channel protein expression. Our data reveal that visceral hypersensitivity is associated with a decrease in SK2 channel activity and protein expression in spinal DH neurons.

## Materials and Methods

### Animals

Sprague-Dawley (SD) rats (220–250 g) were purchased from the Experimental Animal Center of Xuzhou Medical University (Xuzhou, China). One male rat and two female rats were mated to produce offspring. After separation on postnatal day 25, male juvenile rats were grouped into 4 per cage. All rats were housed in a colony with a standard 12–12 h light–dark cycle (lights on at 07:00 AM), constant temperature and humidity (22°C and 50%) with food and water *ad libitum*. Young rats were subsequently assigned to different groups when their body weight reached 200–250 g. All procedures were conducted in accordance with the NIH Guide for the Care and Use of Laboratory Animals (2011) and approved by the Institutional Animal Care and Use Committee at Xuzhou Medical University.

### Reagents

Rabbit anti-SK2/K_Ca_2.2 (APC-028) polyclonal antibody was purchased from Alomone labs (Jerusalem, Israel). Rabbit anti-*c*-Fos mAb (CST-2250s), rabbit NeuN (D4G4O) XP mAb (#24307), and mouse glial fibrillary acidic protein (GFAP) mAb (#3670) were purchased from Cell Signaling Technology (Boston, MA, United States). Mouse anti-β-actin mAb (sc-47778) was purchased from Santa Cruz Biotechnology (Santa Cruz, CA, United States). Goat anti-Iba-1 (ab5076) polyclonal antibody was purchased from Abcam (Cambridge, United Kingdom). Mouse anti-GAPDH (AC001) mAb was purchased from ABclonal Biotechnology (Woburn, MA, United States). Rabbit anti-Tubulin β (AP0064) was purchased from Bioworld Technology (Minneapolis, MN, United States). Alexa Fluor 488 donkey anti-rabbit IgG (H + L) antibody and Alexa Fluor 594 donkey anti-mouse IgG (H + L) were purchased from Life Technologies (Carlsbad, CA, United States). Alkaline phosphatase goat anti-rabbit IgG (ZB-2308), alkaline phosphatase horse anti-mouse IgG (ZB-2310), HRP-labeled goat Anti-Rabbit IgG (H + L) (A0208), HRP-labeled goat anti-mouse IgG (H + L) (A0216), BCA protein assay kit (P0012), sodium dodecyl sulfate (SDS)-polyacrylamide gel electrophoresis (PAGE) sample loading buffer (P0015), BCIP/NBT alkaline phosphatase color development kit (C3206) and BeyoECL Moon kit (P0018FFT) were purchased from Beyotime Institute of Biotechnology (Shanghai, China). Syn-PER^TM^ Synaptic Protein Extraction Reagent (#87793) was purchased from Thermo Fisher Scientific (Waltham, MA, United States).

### Development and Assessment of Visceral Hypersensitivity

**FIGURE 1:**
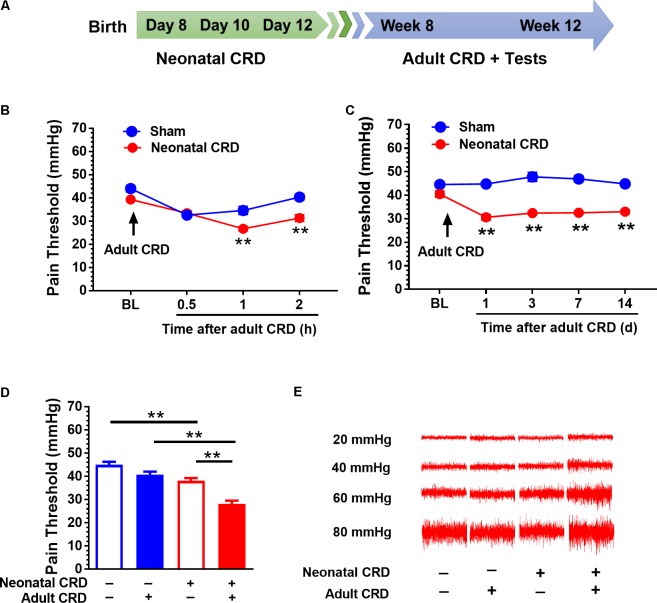
Adult CRD precipitates the visceral hypersensitivity in rats that experienced neonatal CRD. **(A)** Timeline for CRD and behavioral test in rats. Neonatal CRD were conducted on postnatal days 8, 10, and 12. Adult CRD and behavioral test occurred between weeks 8 and 12. **(B)** The pain thresholds of rats that experienced neonatal CRD decreased significantly at 1 and 2 h after re-exposed to adult CRD compared with that in sham group (*p* < 0.01, *n* = 6 per group). **(C)** The pain thresholds of rats that experienced neonatal CRD decreased consistently after re-exposed to adult CRD and lasted for 14 days compared with that in sham group (*p* < 0.01, *n* = 6 per group). **(D)** Neonatal CRD rats presented a significant decrease in pain threshold compared with naïve rats or rats that experienced either neonatal or adult CRD (*p* < 0.01; *n* = 8 per group). **(E)** Representative EMG recordings at 20, 40, 60, and 80 mmHg CRD in rats at different condition. Data are expressed as mean ± SEM. ^∗∗^*p* < 0.01.

The paradigm of development and assessment of visceral hypersensitivity has been well-established (Zhang et al., 2016a). In brief, rats received CRD on postnatal days 8, 10, and 12 by inserting an angioplasty balloon (length, 20.0 mm; diameter, 3.0 mm) into the upper rectum and descending colon (**Figure 1A**). The balloon was distended with 0.3 mL water at a pressure of 60 mmHg for 1 min before deflation and withdrawal. The distention was repeated twice with at least 30 min interval. The sham group rats were not given water inflation to distension balloon. Adult CRD was given to rats at 60 mmHg distention for 60 s 10 times with a 15 s interval. Visceral hypersensitivity was determined by visceral pain threshold in response to adult CRD and external oblique muscle discharge as described previously (Zhang et al., 2016a).

### Assessment of Pain Threshold

Rats were placed in transparent plastic boxes (20 cm × 10 cm × 18 cm) on an elevated plexiglas platform and allowed to habituate to the recording environments for 15–30 min. Rubber balloon in fixed size were inserted into the upper rectum and descending colon before applying different expansion pressures to observe the response of abdominal wall. The pain threshold was defined by the stimulus intensity that evoked a visible contraction of abdominal wall. CRD was applied starting at 10 mmHg. For accuracy, each distension was repeated for three times to calculate an average.

### Intrathecal Catheter Implantation

Intrathecal catheter implantation was similar to that described by Yaksh and Rudy (1976) with some modifications. In brief, rats were anesthetized with 2% pentobarbital sodium (40 mg/kg, i.p.). A 2–3 cm longitudinal incision was made through the skin and muscle on the back of rats to fully expose the L4–5 vertebrae. PE-10 catheter (OD 0.28 mm, OD 0.61 mm) containing 0.9% sterile saline was inserted between L4 and L5. Sterile saline (10 μL) were injected into subarachnoid gap slowly without resistance and cerebrospinal fluid was out from the crevasse, which showed that the catheter was unobstructed. The catheter was secured to the surrounding tissue and the outside end of the catheter was sealed. The surgery wounds were dressed with antibiotic powder and rats were allowed to recover for 5 days. 1-EBIO (300 μg), CyPPA (140 ng), and apamin (5 ng) drugs were given intrathecally in a volume of 10 μL using a microsyringe infusion pump (KDS Scientific, Boston, MA, United States) loaded with a 10 μL Hamilton microsyringe.

### Electrophysiological Recording

Transverse sections of the lower lumbar and upper sacral segment (L4–S4) were taken at 250–300 μm in ice-cold slicing solution (mM): 80 NaCl, 3.5 KCl, 4.5 MgSO_4_, 0.5 CaCl_2_, 1.25 NaH_2_PO_4_, 90 sucrose, 25 NaHCO_3_, and 10 glucose. Artificial cerebral spinal fluid (ACSF) (mM) consisted of 126 NaCl, 2.5 KCl, 1.2 NaH_2_PO_4_, 1.2 MgSO_4_, 26 NaHCO_3_, 10 glucose, and 2.4 CaCl_2_. All solutions were saturated with 95% O_2_ and 5% CO_2_. The slices were incubated in cutting solution at 32°C for 15 min, then transferred to the ACSF at room temperature for at least 1 h prior to a submersion recording chamber. Whole cell patch-clamp recordings were conducted in DH laminae I–II neurons with a glass pipette filled with an internal solution (mM): 10 phosphocreatine-Tris, 10 HEPES, 10 EGTA, 2 ATP-Mg, 0.5 GTP-Na, 115 K gluconate, 20 KCl, 1.5 MgCl_2_; pH was adjusted to 7.2 with KOH (285 mOsm). The resistance was 4–8 MΩ. Whole-cell recoding: cells were held in the current-clamp mode at −60 mV and action potential firings in response to the injection of depolarizing current pulses were recorded with a patch-clamp amplifier (MultiClamp 700A, Axon Instruments, Union City, CA, United States). To measure SK currents, DH neurons were held in voltage-clamp at a holding potential of −60 mV and 100 ms depolarizing pulse to 60 mV, which was used to evoke an outward current. Cell-attached recordings were performed in voltage clamp with the current held around 0 pA. Data acquisition and analysis were performed using Clampex and Clampfit 10 (Axon Instruments, San Jose, CA, United States).

### Immunofluorescent Staining

Rats were deeply anesthetized and transcardially perfused with 0.9% saline (100 mL/100 g), followed by 4% polyformaldehyde in phosphate buffer (100 mL/100 g). Spinal cord was harvested and fixed again in 4% polyformaldehyde overnight and equilibrated in 30% sucrose solution before being sliced at 30 μm thickness with a cryostat (Leica CM1800; Heidelberg, Germany). Selected slices were washed with PBS three times for 10 min and blocked with 10% donkey serum at room temperature for 2 h before being incubated with anti-*c*-Fos antibody (1:6,000), anti-NeuN antibody (1:1,000), anti-GFAP antibody (1:1,000), anti-Iba1 (1:1,000) and anti-SK2 (1:150) at 4°C for 24 h, then was incubated with Alexa Fluor 488 (1:200) or Alexa Fluor 594 (1:200) at room temperature for 2 h. DH regions were visualized with a confocal laser microscope (FV1000, Olympus, Tokyo, Japan).

### Western Blot Analysis

Rats were sacrificed approximately 2 h after adult CRD. The lower lumbar and upper sacral segment (L4–S4) was extracted and lysed with Syn-PER^TM^ Synaptic Protein Extraction Reagent (1 mL/100 mg) containing PMSF. Cell lysis was centrifuged at 8.000 rpm for 10 min at 4°C. The supernatant (whole cell lysis) was collected and further centrifuged at 15,000 rpm for 20 min at 4°C. The supernatant (cytoplasmic fraction) were collected. RIPA lysis buffer (500 μL/200 mg) was added to the pellet (membrane fraction). The sample protein concentration was determined with BCA. Equal amounts of protein were separated by SDS-PAGE gels and transferred to the PVDF membrane. After blockade with 5% non-fat milk for 2 h at room temperature, the PVDF membrane was incubated with anti-SK2 (1:200) anti-GAPDH (1:1,000) or anti-Tubulin β (1:3,000) primary antibody at 4°C overnight. After TBST washing, the PVDF membrane was incubated with AP-conjugated secondary antibody (1:1,000) or HRP-conjugated secondary antibody (1:1,000) for 2 h at room temperature. Protein bands were illuminated using the BCIP/NBT alkaline phosphatase color development kit or BeyoECL Moon kit and captured by using Image ProPlus image analysis system (Media Cybernetics, Inc., Rockville, MD, United States).

### Statistical Analysis

Data are expressed as mean ± SEM. One-way analysis of variance (ANOVA) and repeated measures ANOVA were used. If significance was found, *post hoc* Bonferroni or S–N–K multiple comparisons were used. Independent samples Student’s *t*-test was also used. All statistical tests were conducted using the SPSS 19.0 (IBM, Armonk, NY, United States) software package. *p* < 0.05 was considered statistically significant.

## Results

### Adult CRD Precipitates Visceral Hypersensitivity in Rats That Experienced Neonatal CRD

Rats underwent neonatal CRD on postnatal days 8, 10, and 12, and adult CRD in week 8 to 12 (**Figure 1A**). Pain threshold was recorded 0.5, 1, and 2 h after adult CRD. Neonatal CRD altered the pain threshold (*F*_(1,10)_ = 12.39). Rats that experienced neonatal CRD presented lower pain threshold compared to sham rats at 1 h (*t*_(10)_ = 4, *p* < 0.01) and 2 h (*t*_(10)_ = 4.56*, p* < 0.01) after adult CRD (**Figure 1B**). Pain threshold on days 1, 3, 7, and 14 after adult CRD revealed a significant change (*F*_(1,10)_ = 236.19, *p* < 0.01). Rats that experienced neonatal CRD presented lower pain threshold compared to sham rats on day 1 (*t*_(10)_ = 10.72, *p* < 0.01), day 3 (*t*_(10)_ = 7.6, *p* < 0.01), day 7 (*t*_(10)_ = 10.29, *p* < 0.01), and day 14 (*t*_(10)_ = 12.46, *p* < 0.01) after adult CRD (**Figure 1C**). Rats that experienced both neonatal and adult CRD presented a significant decrease in pain threshold compared with naïve rats or rats that experienced either neonatal or adult CRD (*F*_(3,28)_ = 30.87, *p* < 0.01; **Figure 1D**). **Figure 1E** showed the respective EMG recording to various distension pressure in rats that experienced neonatal and/or adult CRD.

### SK2 Channel Protein in Membrane Fraction and *I*_AHP_ Are Reduced in Rats That Experienced Neonatal CRD

**FIGURE 2:**
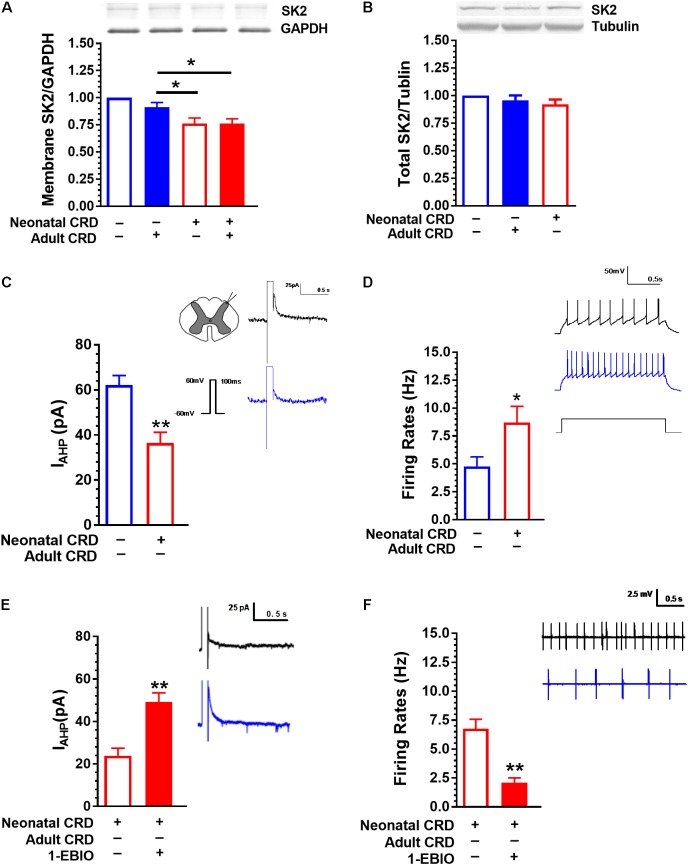
SK2 Channel protein in membrane fraction and *I*_AHP_ are reduced in rats that experienced neonatal CRD. **(A)** Spinal SK2 channel in membrane fraction presented a significant decrease in rats that experienced neonatal and adult CRD compared with that in rats received adult CRD alone (*p* < 0.05, *n* = 3 per group). **(B)** There was no difference in total spinal SK2 channel protein in rats that experienced neonatal or adult CRD as compared to sham rats (*p* > 0.05, *n* = 7 per group). **(C)** The average peak amplitude of *I*_AHP_ in neonatal CRD rats significantly reduced compared with that in naïve rats (*p* < 0.01, *n* = 16–21 neurons, six rats per group). **(D)** The spontaneous neuronal firing rate in spinal DH increased significantly in neonatal CRD rats compared with that in naïve rats (*p* < 0.05, *n* = 9–10 neurons, five rats per group). **(E)** 1-EBIO can increase the amplitude of *I*_AHP_ in neonatal CRD rats (*p* < 0.01, *n* = 10 neurons per group). **(F)** 1-EBIO can decrease the neuronal firing rate in the spinal DH in rats that experienced neonatal CRD (*p* < 0.01, *n* = 10 neurons per group). Data are expressed as mean ± SEM. ^∗^*p* < 0.05, ^∗∗^*p* < 0.01.

Western blot data showed that the spinal SK2 channel protein in membrane fraction was decreased in rats that experienced neonatal with or without adult CRD compared with that in rats only received adult CRD (*F*_(3,8)_ = 13.67, *p* < 0.01; **Figure 2A**). There was no difference in total spinal SK2 protein in rats that experienced either neonatal or adult CRD (*F*_(2,18)_ = 1.18, *p* = 0.33; **Figure 2B**).

Rats that experienced neonatal CRD presented low *I*_AHP_ compared to rats without neonatal CRD. The average peak amplitude of *I*_AHP_ was 62.2 ± 19 pA and 36.43 ± 18.9 pA in naïve and neonatal CRD rats, respectively (*t*_(35)_ = 4.09, *p* < 0.01; **Figure 2C**). Consistently, rats that experienced neonatal CRD presented an increase in spontaneous neuronal firing rates compared to the naïve rats (4.77 ± 0.85 vs. 8.70 ± 1.45; *t*_(17)_ = 2.27, *p* = 0.04; **Figure 2D**). The changes could be reversed by SK channel activator 1-EBIO (100 μM). Incubation of the spinal slice of rats that experienced neonatal CRD. 1-EBIO elevated *I*_AHP_ (23.90 ± 3.51 vs. 49.11 ± 4.37; *t*_(18)_ = −4.53, *p* < 0.01; **Figure 2E**) and decreased the neuron firing rate (6.76 ± 0.82 vs. 2.104 ± 0.40; *t*_(18)_ = 5.11, *p* < 0.01; **Figure 2F**).

### SK Channel Activator 1-EBIO Prevents the Decrease in Pain Threshold and Increase in *c*-Fos Protein Expression in Rats That Experienced Neonatal and Adult CRD

**FIGURE 3:**
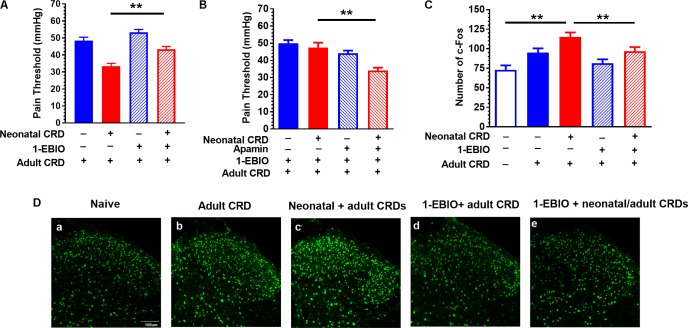
SK channel activator 1-EBIO prevents the decrease in pain threshold and increase in *c*-Fos protein expression in rats that experienced neonatal and adult CRD. **(A)** 1-EBIO prevented the decrease in pain threshold in rats that experienced neonatal and adult CRD (*p* < 0.01, *n* = 6 per group). **(B)** Apamin blocked the effect of 1-EBIO (*n* = 6). **(C)** 1-EBIO can prevent neonatal and adult CRD induced *c*-Fos overexpression (*p* < 0.01, *n* = 6 per group). **(D)** *c*-Fos immunostaining. Scale bar = 100 μm. Data are expressed as mean ± SEM. ^∗∗^*p* < 0.01.

Rats received intrathecal administration of 1-EBIO (300 μg/10 μL) 20 min before adult CRD, and behavioral tests were conducted 2 h after adult CRD. 1-EBIO prevented the decrease in pain threshold in rats that experienced neonatal and adult CRD (*p* < 0.01; **Figure 3A**). The increase in pain threshold induced by 1-EBIO could be further blocked by SK2 channel blocker apamin (5 ng/10 μL) (*p* < 0.01; **Figure 3B**). **Figure 3D** shows the *c*-Fos immunostaining in the spinal DH under different conditions (**Figure 3D**). Quantitative results showed 1-EBIO could prevent neonatal and adult CRD induced increase in *c*-Fos overexpression (*p* < 0.01; **Figure 3C**).

### SK2 Channel Activator CyPPA and Blocker Apamin Affect *I*_AHP_ and Pain Threshold

**FIGURE 4:**
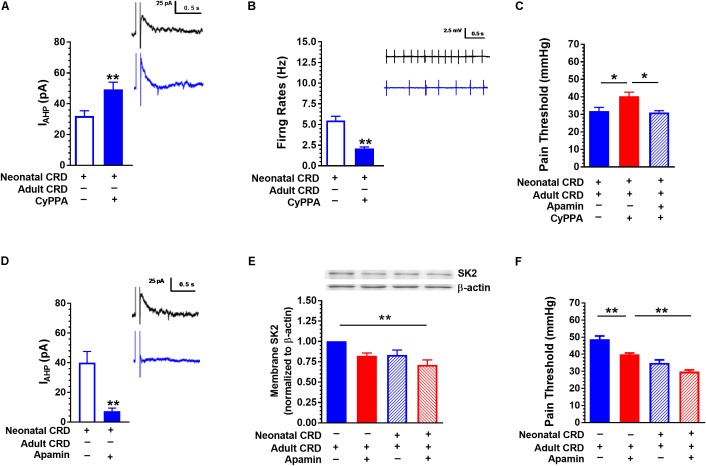
SK2 channel activator CyPPA and blocker apamin affect *I*_AHP_ and pain threshold. **(A)** CyPPA increased the amplitude of *I*_AHP_ in rats that experienced neonatal CRD (*p* < 0.01, *n* = 10 neurons per group). **(B)** CyPPA decreased the neuronal firing rate in the spinal DH in rats that experienced neonatal CRD (*p* < 0.01, *n* = 14 neurons per group). **(C)** CyPPA prevented the decrease in pain threshold in rats that experienced neonatal and adult CRD, which could be prevented by apamin (*p* < 0.01, *n* = 5 per group). **(D)** Apamin decreased the amplitude of *I*_AHP_ in rats that experienced neonatal CRD (*p* < 0.01, *n* = 6 neurons per group). **(E)** Apamin induced a decrease in membrane SK2 channel protein level (*p* < 0.01, *n* = 6 neurons per group). **(F)** Apamin promoted a decrease in pain threshold in rats that experienced neonatal or adult CRD (*p* < 0.01, *n* = 5 per group). Data are expressed as mean ± SEM. ^∗^*p* < 0.05; ^∗∗^*p* < 0.01.

Similar to 1-EBIO, CyPPA (20 μM) increased *I*_AHP_ (32.01 ± 3.42 vs. 49.311 ± 4.63; *t*_(18)_ = −3.01, *p* < 0.01; **Figure 4A**) and suppressed neuronal firing rate (5.49 ± 0.51 vs. 2.09 ± 0.20; *t*_(26)_ = 6.19, *p* < 0.01; **Figure 4B**). Intrathecal CyPPA (140 ng/10 μL) prevented CRD induced decrease in pain threshold, which could be blocked by SK2 channel blocker apamin (5 ng/10 μL) (*p* < 0.05; **Figure 4C**). Apamin (100 nM) decreased *I*_AHP_ current compared with that in control group (40.00 ± 7.54 vs. 7.28 ± 2.15; *t*_(10)_ = 4.18, *p* < 0.01; **Figure 4D**). **Figure 4E** showed the SK2 channel protein in membrane fraction under different conditions. Intrathecal administration of apamin facilitated the decrease in pain threshold caused by CRD (*p* < 0.05; **Figure 4F**).

## Discussion

In this study, we explored the role of spinal SK2 channels in visceral hypersensitivity in a rat model developed by CRD. Rats with visceral hypersensitivity presented a significant decrease in membrane SK2 channel protein and *I*_AHP_, and an increase in neuronal firing rate in the spinal DH. SK2 channel activators 1-EBIO and CyPPA could reverse the decrease in *I*_AHP_ and neuronal firing rate, and prevent the visceral hypersensitivity in CRD rats. SK2 channel blocker apamin reduced *I*_AHP_ and pain threshold in CRD rats. Our results for the first time reported the involvement of spinal SK2 channels in the visceral hypersensitivity, and pharmacological manipulation of SK2 channels may open a new avenue for the treatment of chronic visceral hypersensitivity and pain.

In line with previous reports (Zhang et al., 2016a), we validated that neonatal CRD can induce visceral hypersensitivity and facilitate pain precipitation. Early life is a critical period for the nervous system development. The adverse events in early life can disrupt neuronal development, leading to various endocrine, metabolic, autoimmune, and psychiatric disorders (Amath et al., 2012), as well as increase the vulnerability to stressors (Luthar and Zigler, 1991; Gunnar and Quevedo, 2008). We previously reported that microglial activation in the hippocampus and paraventricular nucleus participates in the etiology of visceral hypersensitivity to neonatal CRD (Zhang et al., 2016a). The spinal DH converges peripheral sensory information and relays to the brain. Abnormalities in the structure and function of DH are observed in neuropathic pain, cancer pain, and other pain disorders (Kawamata and Omote, 1996; Wang et al., 2014; Fu et al., 2016; Medrano et al., 2016). Nociceptive neurons in the spinal DH presented higher excitability and exaggerated sensory reflex to the visceral stimuli in subjects with visceral hypersensitivity (Lu et al., 2007; Hipolito et al., 2015; Wu et al., 2016). Exploration of the molecular mechanism in the spinal DH responsible for visceral hypersensitivity is critical for translational research and drug development.

Our data revealed that membrane SK2 channel protein in spinal DH was decreased in rats that experienced CRD. Consistently, the SK2 channel-mediated *I*_AHP_ was decreased. SK2 channels regulate synaptic transmission, neuronal excitability and firing by allowing K^+^ to efflux in response to increase in the intracellular Ca^2+^ level. The decrease in membrane SK2 channel will facilitate the intrinsic neuronal excitability and synaptic transmission, which may induce visceral hypersensitivity and pain. Moreover, SK2 channel activators 1-EBIO and CyPPA were found to prevent the precipitation of visceral hypersensitivity and pain, which further supports the hypothesis that SK2 channels participate in the pathogenesis of visceral hypersensitivity and pain. *c*-Fos is a proto-oncogene that is expressed within some neurons following depolarization. *c*-Fos expression might be used as a marker for neuronal activity throughout the neuraxis following peripheral stimulation. We found an increase in *c*-Fos positive staining in the spinal DH, which was in line with the increase in spinal neuronal firing in rats that experienced visceral hypersensitivity. 1-EBIO prevented the increase in *c*-Fos protein expression in spinal DH in rats that experienced CRD further support that activation of SK2 channels presents an inhibitory effect.

The mechanism underlying the decrease in membrane SK2 channel protein remains unknown. Activation of cyclic AMP-dependent protein kinase (PKA) with forskolin causes a dramatic decrease in surface localization of the SK2 channel subunit expressed in COS7 cells due to direct phosphorylation of the SK2 channel intracellular domain (Ren et al., 2006; Abiraman et al., 2016). The internalization of synaptic SK channels by PKA enhances excitatory synaptic transmission and plasticity in the Amygdala (Faber et al., 2008). Blocking PKA or PKA target domain in SK2 channels blocks the internalization of SK2 channels after long-term potentiation (LTP) induction at Schaffer collateral-CA1 synapses (Lin et al., 2008). The cAMP-PKA pathway augments presynaptic neurotransmitter synthesis and vesicular transport, maintains DRG neuronal hyperexcitability by phosphorylating key transcription factors and synaptic vesicle proteins (King et al., 2005; Tumati et al., 2011; Zhu et al., 2014; Shao et al., 2016). These findings suggest that CRD causes the internalization of SK2 channels by increasing cAMP-PKA activity in spinal DH.

SK channels are specifically involved in the medium afterhyperpolarization potential (mAHP) following single or multiple action potentials in neurons, affecting the intrinsic excitability of neurons, synaptic transmission and pain sensation (Bahia et al., 2005; Pedarzani et al., 2005; Lu et al., 2009). In an animal model of nociception, the AHP is down-regulated in DRG cells and reticulospinal neurons after nerve injury (McClellan et al., 2008). In this study, we found that SK2 channel protein in membrane fraction was decreased, which may result in the reduction in the amplitude of *I*_AHP_ contributing to an increase in neuronal excitability, e.g., increase in neuronal firing rate.

Besides SK2 channels, SK3 channel may also participate the development of visceral pain. Similar to SK2 channels, high level of SK3 channel positive staining are expressed in the DH of spinal cord. Particularly, dense fiber staining was observed in the laminae I and II (Sailer et al., 2004; Hipólito et al., 2015). Our data confirm this finding (Supplementary Figure 2). Moreover, neonatal and/or adult CRD did not alter the SK3 channel protein expression in the spinal cord (Supplementary Figure 3). Considering the limitation of SK3 channel selective activator/blocker, the role of SK3 channel in the etiology of visceral pain need further exploration.

Neuroinflammation participates in the development of visceral hypersensitivity and pain. We previously reported microglial activation and increase in proinflammatory cytokines facilitate the development of visceral hypersensitivity in rats that experienced CRD (Zhang et al., 2016a). *KCNN1-3* genes are expressed in microglia (Schlichter et al., 2010). SK channels regulates microglial function and neuroninflammation (Armstrong et al., 2005; Schlichter et al., 2010; Dolga and Culmsee, 2012). For example, the SK3 channel mRNA is expressed in microglia in the rat striatum (Schlichter et al., 2010), substantia nigra pars compacta (Armstrong et al., 2005) of adult rat and mouse brains. SK3 channel blockade decreased the activity of p38 mitogen-activated protein kinase (MAPK) in microglia cells and attenuated tyrosine-nitrated proteins in the neurons exposed to activated microglia (Schlichter et al., 2010). We found that SK2 channels are co-labeled with GAFP and Iba-1 at the layer I and II of the spinal DH, supporting the involvement of SK2 channels in the modulation of neuroinflammation (Supplementary Figure 1).

In this study, we explored the participation of SK2 channels in spinal DH on visceral hypersensitivity in rats. Our data reveals that rats with visceral hypersensitivity present a decrease in the number and function of membrane SK2 channels in the spinal DH. Pharmacological activation of SK2 channels can prevent the precipitation of visceral hypersensitivity, and blockade of SK2 channel can facilitate the visceral hypersensitivity. Pharmacological manipulation of SK2 channels may open a new avenue for the treatment of visceral hypersensitivity and pain.

## Author Contributions

Y-MZ, GZ, and RS conceived and planned the experiments. J-SZ, YS, LD, S-TH, and RH carried out the experiments. All authors contributed to data analysis and manuscript preparation.

## Conflict of Interest Statement

The authors declare that the research was conducted in the absence of any commercial or financial relationships that could be construed as a potential conflict of interest.
