# Supplementary material for: Small-Conductance Ca2+-Activated K+ Channel 2 in the Dorsal Horn of Spinal Cord Participates in Visceral Hypersensitivity in Rats
Source: Front Pharmacol. 2018 Aug 3;9:840. doi: 10.3389/fphar.2018.00840 (PMC6085475; doi:10.3389/fphar.2018.00840)
Supplement: Supplementary file 1 [file Data_Sheet_1.docx]

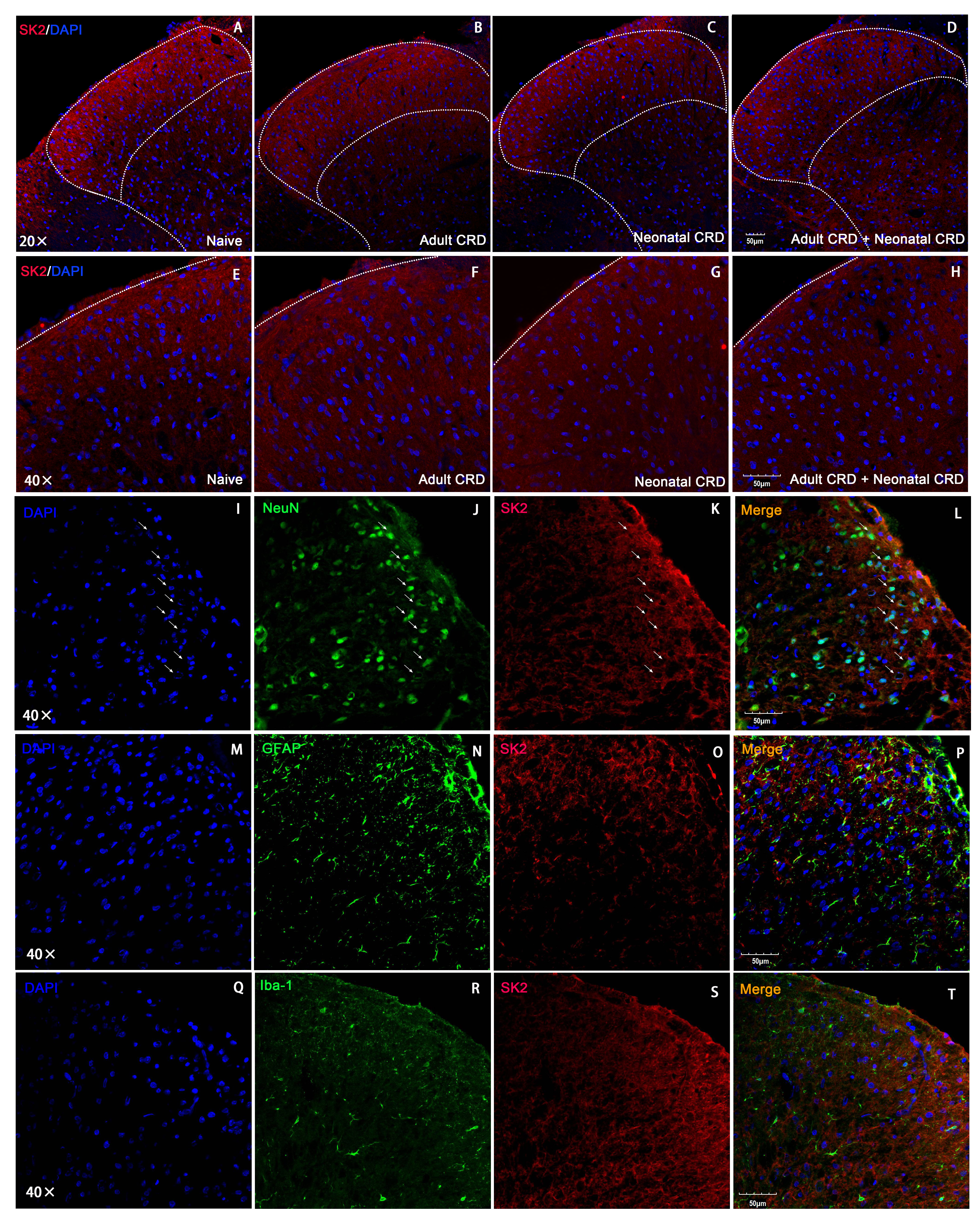


**SUPPLEMENTARY FIGURE 1**. Immunofluoresence staining of SK2 channels in the spinal DH of rats. (A-H) SK2 channel positive stainings were located at the layer I and II of the spinal DH. (I-L) SK2 channel protein was co-labeled with NeuN. (M-P) A limited number of SK2 channel protein was co-labeled with GFAP. (Q-T) A limited number of SK2 channel protein was co-labeled with Iba-1. Low magnification: 20×, High magnification 40×; Scale bars 50 μm.


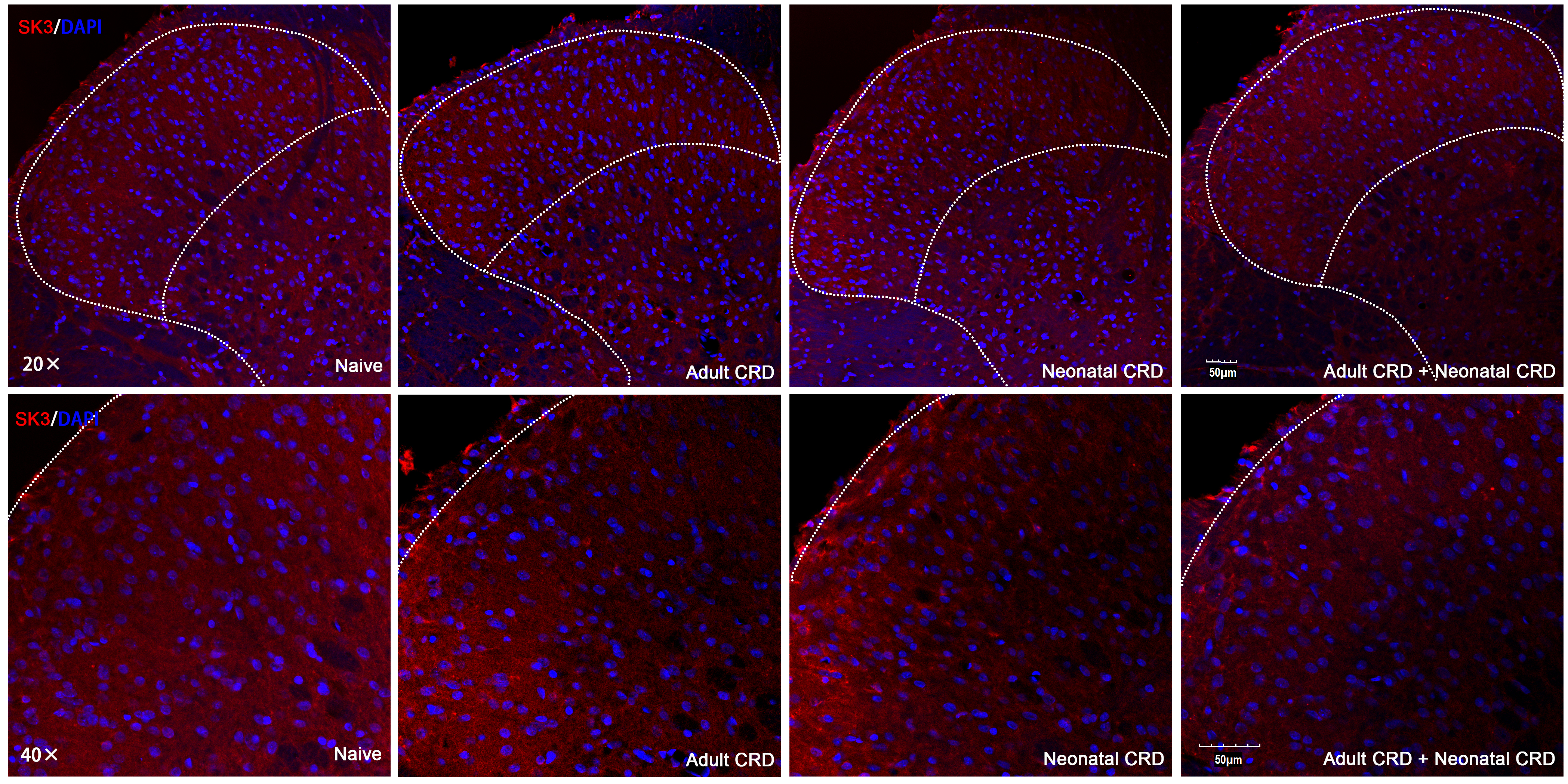


**SUPPLEMENTARY FIGURE 2**. Distribution of SK3 channel protein in the spinal DH of rats that experienced neonatal and/or adult CRD. Scale bars 50 μm.

**SUPPLEMENTARY FIGURE 3**. Spinal SK3 channel protein was not changed in rats that experienced neonatal and/or adult CRD. Data are expressed as mean ± SEM. n = 3 per group.
